# Prevalence and overlap of Disease Management Program diseases in older hospitalized patients

**DOI:** 10.1007/s10433-017-0412-9

**Published:** 2017-02-04

**Authors:** Helle Gybel Juul-Larsen, Janne Petersen, Ditte Maria Sivertsen, Ove Andersen

**Affiliations:** 1 Optimed, Clinical Research Centre, Amager Hvidovre Hospital, University of Copenhagen, Kettegaard Allé 30, 2650 Hvidovre, Denmark; 2 Department of Clinical Medicine, University of Copenhagen, Blegdamsvej 3B, 2200 Copenhagen N, Denmark; 3 Section of Biostatistics, Department of Public Health, University of Copenhagen, Øster Farimagsgade 5, 1014 Copenhagen K, Denmark

**Keywords:** Disease management program, Older hospitalized patients, Multimorbidity

## Abstract

Many countries, like Denmark, have tailored Disease Management Programs (DMPs) based on patients having single chronic diseases [defined institutionally as “program diseases” (PDs)], which can complicate treatment for those with multiple chronic diseases. The aims of this study were (a) to assess the prevalence and overlap among acutely hospitalized older medical patients of PDs defined by the DMPs, and (b) to examine transitions between different departments during hospitalization and mortality and readmission within two time intervals among patients with the different PDs. We conducted a registry study of 4649 acutely hospitalized medical patients ≥65 years admitted to Copenhagen University Hospital, Hvidovre, Denmark, in 2012, and divided patients into six PD groups (type 2 diabetes, chronic obstructive pulmonary disease, cardiovascular disease, musculoskeletal disease, dementia and cancer), each defined by several ICD-10 codes predefined in the DMPs. Of these patients, 904 (19.4%) had 2 + PDs, and there were 47 different combinations of the six different PDs. The most prevalent pair of PDs was type 2 diabetes with cardiovascular disease in 203 (22.5%) patients, of whom 40.4% had an additional PD. The range of the cumulative incidence of being readmitted within 90 days was between 28.8% for patients without a PD and 46.6% for patients with more than one PD. PDs overlapped in many combinations, and all patients had a high probability of being readmitted. Hence, developing strategies to create a new generation of DMPs applicable to older patients with comorbidities could help clinicians organize treatment across DMPs.

## Introduction

The prevalence of chronic diseases increases with age (van den Akker et al. 1998; Fortin et al. 2005; Denton and Spencer 2010) and systematic reviews have found a prevalence of multimorbidity (multiple chronic diseases) of up to 98% in persons aged 60 or older (Marengoni et al. 2011; Fortin et al. 2012). The high number of people with multimorbidity puts pressure on the existing hospital system, which in Denmark as in many other countries is organized into specialized wards according to organ systems. Patients with diseases in more than one organ system risk incoherent trajectories in association with the hospitalization because the coordination among different departments and different parts of the health service may be perceived as problematic (Boyd et al. 2007; Boult and Wieland 2010). Thus, prioritizing collaboration and communication around patients with chronic conditions is important.

To improve management of chronic diseases, the Danish Health and Medicines Authority has recommended Disease Management Programs (DMPs) tailored to Danish health care (Danish Health and Medicines Authority 2012a), similar to several other countries (Lugtenberg et al. 2011). The DMPs are standardized descriptions of the multidisciplinary, multisectional, coordinated and evidence-based healthcare work. This work includes prevention, diagnosis, treatment, rehabilitation and follow-up, cooperation and coordination between the acute and primary care settings based on a specific patient group (Danish Health and Medicines Authority 2012a).

The DMPs address single diseases which are in accordance with the focus on treatment of single diseases in medicine (Tinetti and Fried 2004). Greater attention to multiple chronic diseases (multimorbidity) (Boyd and Fortin 2011) is warranted, and several countries have included multimorbidity in their DMPs (Vitry and Zhang 2008; Lugtenberg et al. 2011). Multimorbidity is often discussed in general without concrete actions (Lugtenberg et al. 2011), and the evidence for treatment and rehabilitation still relies on a single disease concept (Danish Health and Medicines Authority 2012a). Most studies are designed for examining single conditions, and individuals with multimorbidity are therefore often excluded (Starfield 2001; Fortin et al. 2006). International studies problematize the DMPs’ single disease focus when treating patients with multimorbidity, stating that DMPs provide limited guidance on the combined use of treatments (Tinetti and Fried 2004; Vitry and Zhang 2008; Boult and Wieland 2010; Boyd and Fortin 2011; Lugtenberg et al. 2011; Cox et al. 2011; Mutasingwa et al. 2011; Hughes et al. 2013). Several professional societies and researchers around the world have started developing guidelines or DMPs for older patients with multimorbidity (Fabbri et al. 2012; Uhlig et al. 2014; Weiss et al. 2014; Bernabeu-Wittel et al. 2014), but the complexity of the different treatment regimens and the interactions between the DMPs makes the work difficult. More knowledge is needed about how the patients’ different chronic diseases occur together and the trajectories for these patients.

In Denmark, DMPs have primarily been implemented in the primary care setting and in outpatient clinics. Improving patient care requires implementing coordinated health care across all sectors to increase our understanding of complex medical patients and their management. As it is today, however, hospitals are organized in specialized wards based on single diseases. Therefore, it is important to investigate the extent of multimorbidity (based on DMPs) in the hospital setting and explore the trajectories of these patients. The aims of this study thus were (a) to study the prevalence and overlap of program diseases (PDs) defined by the DMPs among acutely hospitalized older medical patients, and (b) to examine transitions among different departments during hospitalization and mortality and readmission within two time intervals in patients with different PDs.

## Design and methods

### Setting

In Denmark, the publicly funded healthcare system covers all primary and specialist services uniformly for all citizens. Amager and Hvidovre Hospital, University of Copenhagen, covers 10 municipalities with approximately 460,000 citizens and has approximately 14,000 medical admissions each year. Of these, 85% are acute. The emergency department (ED) at Amager and Hvidovre Hospital consists of a traditional ED and a medical unit, where patients referred by general practitioners or by ambulance due to an emergency call can be hospitalized for up to 3 days before discharge or transfer to a specialized medical ward. All Danish citizens have a unique personal identification number, the Central Personal Register number (CPR number) (Pedersen 2011). Because of the CPR number, linkage at the individual level among nationwide and local registries is feasible.

### Study population

Because of the large number of patients hospitalized acutely, the study population included all medical patients with a Danish CPR number, aged ≥ 65 years old, who were acutely admitted to the medical unit at the ED at Amager and Hvidovre Hospital from January 1, 2012, to December 31, 2012. Patients were divided into eight groups: one group with no PD, six groups of patients, respectively, having only one of the six PDs [type 2 diabetes, chronic obstructive pulmonary disease (COPD), cardiovascular or musculoskeletal disease, dementia or cancer], and a group of patients with two or more PDs.

### Data collection

Data were collected from the Danish National Patient Registry, a nationwide population registry in Denmark (Lynge et al. 2011), from the Danish Civil Registration System (Pedersen 2011), and from the local registry of Amager and Hvidovre Hospital, University of Copenhagen. The World Health Organization’s updated International Classification of Diseases 10th edition (ICD-10) classification system was used to define PDs. The system is divided into 21 chapters representing different organ systems and categories of health problems (World Health Organization 2014).

### Outcomes

PD: A PD is predefined by the Danish Health and Medicines Authority by the presence of at least one of the following ICD-10 codes: type 2 diabetes (E10–E14), COPD (J44), cardiovascular disease (I20–I21, I25.1 and I50), musculoskeletal (G550–G553, G558, L88, L71, L97, M431, M471, M472, M478E, M480, M482, M485B, M510–M514, M511F, M533, M533B, M539, M543–M545 and S336A), dementia (G30.0, G30.1, G30.8, G31.0B, G31.8, G31.8E, G31.9, I69.4 and I69.3) and cancer (C00–C99) according to the Danish DMPs (Capital Region, Denmark 2009a, b, 2011, 2012a, b; Danish Health and Medicines Authority 2012b). ICD-10 codes were extracted from the Danish National Patient Registry based on both the patient’s first acute hospital admission in 2012 and a 10-year prevalence of the ICD-10 codes registered prior to the index admission based on recommendations in Schram et al. (2008).

Transitions during hospitalization: Information regarding the patients’ hospital departments during hospitalization was recorded from the local registry of Amager and Hvidovre Hospital, based on data from the first acute hospital admission in 2012. One transition was defined as a transition from the medical unit at the ED to a specialized ward, and two or more transitions were defined as a transition from the medical unit at the ED to a specialized ward and an additional transition to another specialized ward.

#### Mortality

Time of death was recorded from the Danish Civil Registration System and from admission and discharge dates from the Danish National Patient Register. Time to death was recorded in three different time intervals: during hospitalization, to reflect the quality of care during hospitalization; 7 days after admission, to reflect the quality of care and the discharge process; and 90 days after discharge, to reflect the patients’ general health.

#### Readmissions

Admission and discharge dates were recorded from the Danish National Patient Register. To avoid overestimating the number of readmissions, a hospital readmission was defined as an acute admission more than 4 h after discharge. Both acute readmission dates up to 7 and 90 days after discharge were obtained from the register. An interval of 7 days was chosen to reflect the quality of care in the previous hospitalization and discharge process, and 90 days was chosen to reflect the patients’ general health.

### Descriptive data

From the Danish Civil Registration System, data were collected on age and sex. From the Danish National Patient Register, data were collected on ICD-10 diagnosis codes (acute and chronic) registered for the first acute hospital admission in 2012; on length of stay (LOS), based on data from the first acute hospital admission in 2012; on ICD-10 disease categories, which were categorized according to 17 of the 21 ICD-10 chapters (ICD-10 chapters XV “Pregnancy, childbirth and the puerperium,” XVI “Certain conditions originating in the perinatal period,” XVII “Congenital malformations, deformations and chromosomal abnormalities,” and XIX “Injury, poisoning and certain other consequences of external causes,” were not included because of the lack of relevance); and on acute hospitalization within 6 months prior to the index admission to reflect patients’ general health.

The study was approved by the Danish Data Protection Agency (FSEID-00000882). No approval from the National Committee on Health Research Ethics was needed because only registries were used.

### Statistical analysis

Data are presented as numbers and percentages or as medians with a corresponding interquartile range (IQR). To examine for differences between patients with 2 + PDs and the other groups, we used a multinomial logistic regression model for the following variables: age (adjusted for sex), sex (adjusted for age), ICD-10 disease categories (adjusted for age and sex), and acute hospitalization within 6 months (adjusted for age and sex). All analyses were carried out to estimate odds ratios (ORs) with 99% confidence intervals (CIs). The Kruskal–Wallis test was used to test for differences between patients with 2 + PDs and the other groups regarding LOS. A multinomial logistic regression model (adjusted for age and sex) was also used to examine whether PDs were associated with transitions between different departments during hospitalization.

A Cox proportional hazards model was used to test for the difference between patients with 2 + PDs and the other groups in time to readmission (within 7 days and at 90 days after discharge) and to test for the difference in time to death (during hospitalization, within 7 days after admission and at 90 days after discharge); the analyses were adjusted for age, sex, and acute hospitalization within the last 6 months. A cumulative incidence function was used to plot time to death, and a cumulative incidence function with death as competing event was used to plot time to readmission for the PDs.

The statistical analyses were conducted using the SAS 9.3 software package for Windows. Plots were created in R version 3.1.0. The level of significance was set at 0.01 to account for multiple testing, and all statistical tests were two-tailed.

## Results

In 2012, a total of 4649 patients were admitted acutely to the medical unit at the ED at Amager and Hvidovre Hospital, and their characteristics are shown in Table 1. Of these patients, 904 (19.4%) had 2 + PDs, 1795 (38.6%) had one PD, and 1950 (41.9%) patients did not have a PD. Moreover, in comparing patients with 2 + PDs with the other groups, multinomial regression models showed that patients with the type 2 diabetes PD (OR 0.98, CI_99%_ 0.96–1.00) were younger than patients with 2 + PDs and patients with the dementia PD were older than patients with 2 + PDs (OR 1.06, CI_99%_ 1.04–1.08). There were more men in the 2 + PD group than among patients with the COPD PD (OR 1.57, CI_99%_ 1.16–2.13) and more women in the 2 + PD group than among patients with the cardiovascular PD (OR 1.80, CI_99%_ 1.27–2.55). Overall, the LOS was short, with a median between one and 2 days for all groups except the cancer PD, which had a median LOS of 4 days. Patients with 2 + PD had a higher LOS than patients with no PD (*p* = 0.001) and patients with the cardiovascular PD (*p* = 0.008), but a shorter LOS than patients with the cancer PD (*p* = 0.001). The risk of being registered with three or more ICD-10 disease categories was significantly higher for patients with one or 2 + PDs compared with patients not having a PD. Patients with 2 + PDs had a higher risk of having been acutely hospitalized within the last 6 months prior to the index admission than patients with no PD (OR 3.60, CI_99%_ 2.88–4.52), type 2 diabetes PD (OR 2.23, CI_99%_ 1.57–3.17), COPD PD (OR 1.78, CI_99%_ 1.32–2.41), cardiovascular PD (OR 2.64, CI_99%_ 1.82–3.38), musculoskeletal PD (OR 1.99, CI_99%_ 1.16–3.43) and dementia PD (OR 1.46, CI_99%_ 1.02–2.08).

**Table 1:** Characteristics of the study population

Variable	Total		No PD		Program diseases													
					Type 2 diabetes		COPD		Cardiovascular		Musculoskeletal		Dementia		Cancer		2 + PDs	
*N*	4649		1950		352		484		314		116		292		237		904	
Age, median (IQR)	78.7	(71; 85)	78.5	(71; 86)	76.5	(70; 84)	77.6	(72; 84)	78.3	(73; 86)	81.7	(75; 86)	84.3	(78; 89)	77.4	(71; 85)	78.7	(73; 84)
Female	2653	(57.1)	1164	(59.7)	180	(51.1)	313	(64.7)	130	(41.4)	73	(62.9)	178	(61.0)	119	(50.2)	496	(54.9)
Diagnoses per person^a^, median (IQR)	2	(1; 2)	1	(1; 2)	2	(1; 3)	2	(1; 3)	1	(1; 2)	1	(1; 2)	2	(1; 3)	2	(1; 2)	2	(1; 3)
LOS^#^, median (IQR)	2	(1; 6)	1	(1; 5)	2	(1; 6)	2	(1; 7)	2	(1; 5)	1	(0; 6)	2	(1; 5)	4	(1; 10)	2	(1; 7)
ICD-10 disease categories with 10-year history																		
0–1	533	(11.9)	457	(23.4)	11	(3.1)	48	(9.9)	21	(6.7)	7	(6.0)	4	(1.4)	5	(2.1)	0	(0)
2–4	2039	(43.9)	1062	(54.5)	174	(49.4)	229	(47.3)	162	(51.6)	50	(43.1)	87	(29.8)	106	(44.7)	169	(18.7)
5+	2057	(44.3)	431	(22.1)	167	(47.4)	207	(42.8)	131	(41.7)	59	(50.9)	201	(68.8)	126	(53.2)	735	(81.3)
Acute hospitalization 6 month prior to index admission	1426	(30.7)	394	(20.2)	101	(28.7)	161	(33.3)	83	(26.4)	37	(31.9)	117	(40.1)	102	(43.0)	431	(47.7)

Results are presented as numbers and percentages unless otherwise specified

*PD* program disease, *N* number, *IQR* interquartile range, *COPD* chronic obstructive pulmonary disease, *LOS* length of stay

^a^Data are based on the first hospitalization in 2012. ICD-10 disease categories = number of chapters in the updated International Classification of Diseases 10th edition

**Fig. 1:**
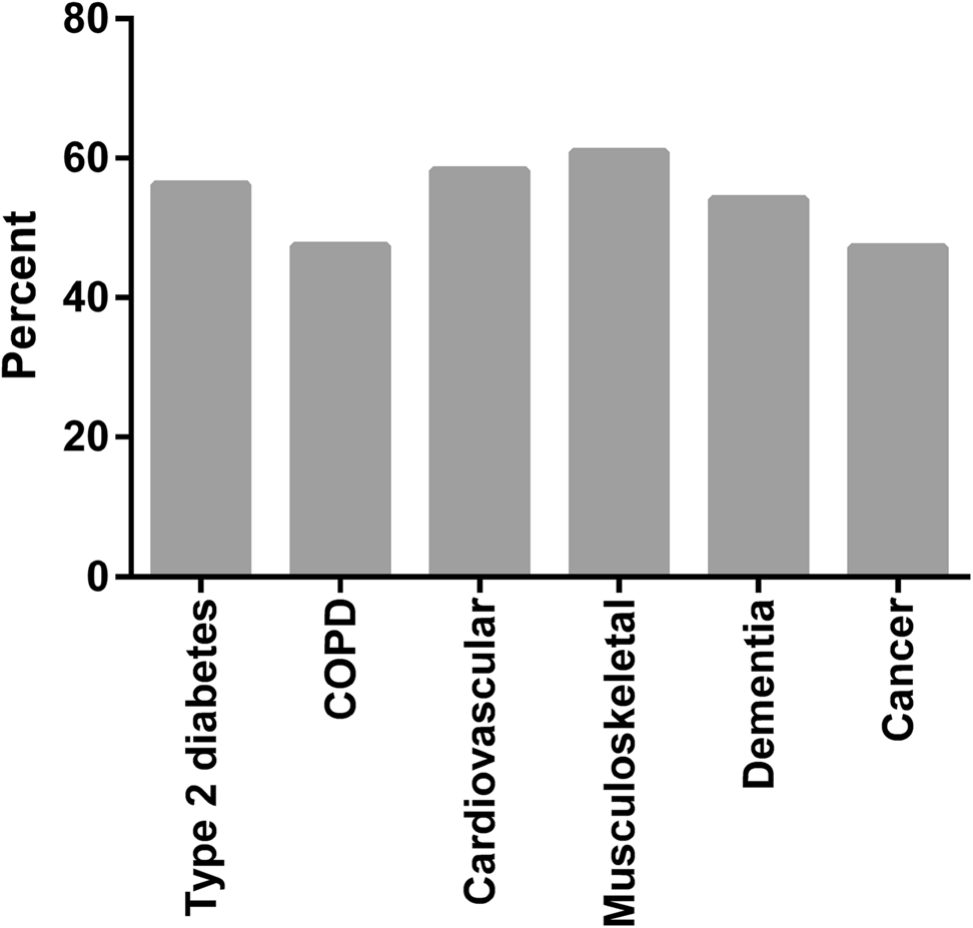
Pecentage of patients having two or more program diseases among patients with at least one program disease. Number of patients with the program disease: diabetes type 2 (*N* = 804), COPD (*N* = 920), cardiovascular (*N* = 751), Musculoskeletal (*N* = 297), dementia (*N* = 636) and cancer (*N* = 449). *COPD* chronic obstructive pulmonary disease

There were 47 different combinations of overlap of PDs with frequencies between 0.1 and 13.4% among the 904 patients with 2 + PDs. Figure 1 shows the proportion of patients having 2 + PDs within each of the PDs. The highest proportion was found for the musculoskeletal PD, with 60.9% of the 297 patients having 2 + PDs, and the lowest proportion was found for the cancer PD, with 47.2% of 449 patients having 2 + PDs. The four most prevalent pairs of PDs among the 904 patients who had more than one PD are shown in Table 2. The most prevalent pair was the type 2 diabetes with the cardiovascular PD, with 203 (22.5%) patients, and 40.4% of those patients had an additional PD.

**Table 2:** The four most prevalent pairs of program diseases among patients with more than one program disease (*N* = 904)

		The four most prevalent pairs of program diseases		Patients with one or more additional PD	
		*N*	%	*N*	%
1	Type 2 diabetes + cardiovascular	203	22.5	82	40.4
2	COPD + cardiovascular	164	18.1	82	50.0
3	Type 2 diabetes + COPD	150	16.6	89	59.3
4	COPD + dementia	137	15.1	68	49.6

*COPD* chronic obstructive pulmonary disease

A total of 2511 (54.0%) patients were discharged directly from the medical unit at the ED, 1733 (37.3%) patients experienced one transition from the medical unit at the ED to a specialized medical department, and 405 (8.7%) patients experienced 2 + transitions between departments (Table 3). Patients belonging to the COPD PD group had a higher risk of being transferred two or more times during hospitalization than patients with 2 + PD (OR 1.99, CI_99%_ 1.19–3.33).

**Table 3:** Transitions during hospitalization, mortality and readmission for the population

Variable	Total		No PD		Program diseases													
					Type 2 diabetes		COPD		Cardiovascular		Musculoskeletal		Dementia		Cancer		2 + PDs	
N	4649		1950		352		484		314		116		292		237		904	
Transitions during hospitalization																		
0	2511	(54.0)	1109	(56.9)	200	(56.8)	240	(49.6)	150	(47.8)	72	(62.1)	160	(54.8)	103	(43.5)	477	(52.8)
1	1733	(37.3)	665	(34.1)	126	(35.8)	185	(38.2)	135	(43.0)	39	(33.6)	106	(36.3)	110	(46.4)	367	(40.6)
2+	405	(8.7)	176	(9.0)	26	(7.4)	59	(12.3)	29	(9.2)	5	(4.3)	26	(8.9)	24	(10.1)	60	(6.64)
Died during hospitalization	310	(6.7)	87	(4.5)	18	(5.1)	35	(7.2)	13	(4.1)	3	(2.6)	18	(6.2)	51	(21.5)	85	(9.4)
Mortality																		
Within 7 days from admission	188	(4.0)	57	(2.9)	10	(2.8)	21	(4.3)	7	(2.2)	0	(0)	17	(5.8)	27	(11.4)	49	(5.4)
Within 90 days from discharge^a^	499	(11.5)	151	(8.1)	33	(9.9)	40	(8.9)	28	(9.3)	10	(8.9)	55	(20.1)	59	(31.7)	123	(15.0)
Readmissions^a^																		
Within 7 days from discharge	423	(9.7)	143	(7.7)	35	(10.5)	51	(11.4)	37	(12.3)	11	(9.7)	36	(13.1)	14	(7.5)	96	(11.7)
Within 90 days from discharge	1568	(36.1)	536	(28.8)	127	(38.0)	196	(43.7)	107	(35.5)	39	(34.5)	104	(38.0)	77	(41.4)	382	(46.6)

Results are presented as numbers and percentages unless otherwise specified

*PD* program disease, *N* number, *COPD* chronic obstructive pulmonary disease

^a^Data are based on the patients who survived to be discharged (total *N* = 4339)

**Fig. 2:**
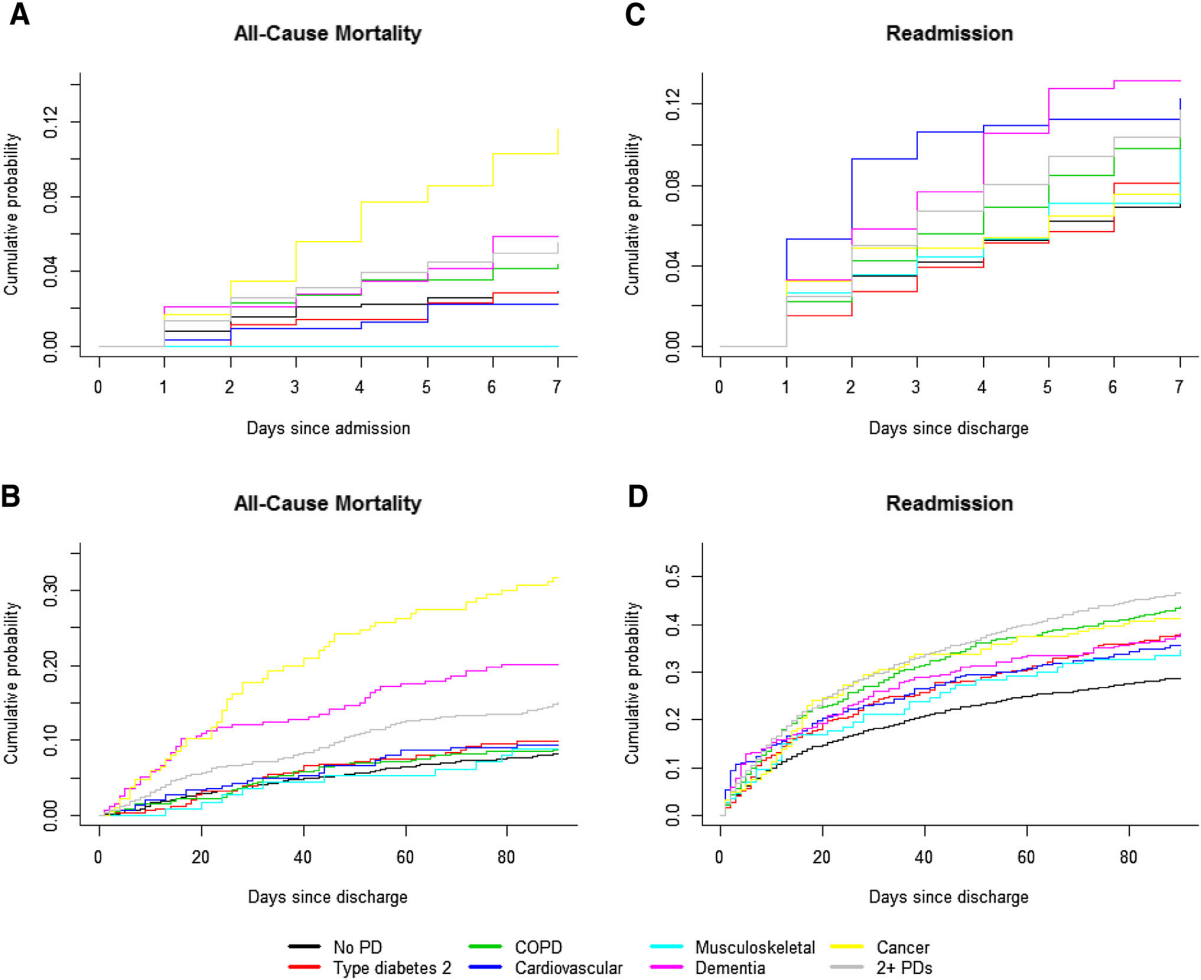
Cumulative incidence plot of time to event for all-cause mortality within 7 days of admission (**a**) and within 90 days from discharge (**b**) and readmission within seven (**c**) and 90 days from discharge (**d**) according to program disease. *PD* program disease, *COPD* chronic obstructive pulmonary disease

In total, 310 (6.7%) patients died during hospitalization (Table 3). When comparing patients with 2 + PDs with the other groups, patients with no PD had a lower risk of dying (hazard ratio (HR) 0.64, CI_99%_ 0.43–0.95), and patients with the cancer PD had a higher risk (HR 1.76, CI_99%_ 1.11–2.78). The all-cause mortality within 7 days from admission was higher only for patients with the cancer PD (HR 2.29, CI_99%_ 1.23–4.24) compared to patients with 2 + PDs (Fig. 2a).

Of the 4339 patients who survived the hospitalization, 499 (11.5%) died within 90 days after discharge (Table 3). The difference between the PDs is depicted in Fig. 2b. When comparing patients with 2 + PDs with the other groups, patients with no PD (HR 0.62 CI_99%_ 0.45–0.86) and patients with the COPD PD (HR 0.62, CI_99%_ 0.39–1.00) had a lower risk of dying, and patients with the cancer PD had a higher risk (HR 2.51, CI_99%_ 1.67–3.78).

In total, 423 out of 4339 (9.7%) patients were readmitted within 7 days from discharge of the patients who survived the hospitalization (Table 3). The adjusted Cox regression model showed no difference between patients with 2 + PD and any of the other groups. The cumulative incidence was 11.7% for patients with 2 + PDs and 7.7% for patients with no PD (Fig. 2c).

Overall, 1568 out of 4339 (36.1%) patients who survived the hospitalization were readmitted within 90 days of discharge of the patients (Table 3). The cumulative probability was 46.6% for patients with 2 + PD and 28.8% for patients with no PD. When comparing patients with 2 + PDs with the other groups, patients with no PD (HR 0.53, CI_99%_ 0.45–0.63), type 2 diabetes PD (HR 0.74, CI_99%_ 0.57–0.96) and cardiovascular PD (HR 0.71, CI_99%_ 0.53–0.94) all had a lower risk of readmission (Fig. 2d).

## Discussion

We have identified no other study that has questioned the single disease focus when treating older patients by exploring the prevalence and overlap of PDs in a large population of older medical acutely hospitalized patients. The major findings of this study are that (a) 19.4% of the patients had 2 + PDs with overlaps appearing in 47 different combinations at a mostly low prevalence, the most prevalent combination being type 2 diabetes and the cardiovascular PD. However, 40.4% of the patients with this combination had at least one more PD; (b) in total, 54.0% were discharged directly from the ED. Patients with 2 + PDs had a higher risk of both dying during hospitalization and dying after hospitalization than patients without a PD. Patients with 2 + PDs had a higher risk of readmission within 90 than patients with no PD, type 2 diabetes PD and cardiovascular PD. No differences were seen for readmission within 7 days; (c) patients with the cancer PD had the lowest proportion of patients with 2 + PDs, the highest LOS, and higher risks of dying or being readmitted within seven and 90 days.

In line with previous studies (Fortin et al. 2005; Marengoni et al. 2009; van den Bussche et al. 2011; Marengoni et al. 2011; Kirchberger et al. 2012), we found a high prevalence of patients with more than one PD. Having two or more PDs was frequent, with overlaps appearing in many combinations with a mostly low prevalence. This result is in agreement with other studies demonstrating a similar prevalence of combinations of chronic conditions despite a more extended list of chronic diseases (Marengoni et al. 2009; Kirchberger et al. 2012). The problem with overlapping PDs is the complex treatment and self-care follow-up regimes for hypothetical patients following more than one clinical guideline, as illustrated by Boyd et al. (Boyd et al. 2005) and Hughes et al. (2013). A recent study has shown that one chronic disease can adversely affect the management of another chronic disease and that the physician together with the patient must weight the pros and cons of several registered treatments (Søndergaard et al. 2015). These results highlight the question of whether or not DMPs should be constructed based on single diseases. Furthermore, polypharmacy and drug interactions are a major concern when following two or more DMPs (Boyd et al. 2005; Hughes et al. 2013). Polypharmacy may be necessary but is associated with severe adverse events (Koper et al. 2013). In addition, the use of potentially inappropriate medications is common among older persons and associated with low functional capacity and low health-related quality of life (Jensen et al. 2014). The difficulties in developing DMPs relevant to patients with multimorbidity lie in part in the complexity of the different recommendations for the medical treatment for the different chronic diseases (Fabbri et al. 2012). Resolving these difficulties requires knowledge about which chronic diseases occur together so that the recommendations can take into account possible drug interactions. Several studies have examined patterns of multimorbidity and found between three and five such patterns (Marengoni et al. 2009; Schäfer et al. 2010; García-Olmos et al. 2012; Kirchberger et al. 2012; Prados-Torres et al. 2012; Freund et al. 2012). Knowledge and understanding of disease patterns occurring in multimorbidity can provide information that may support existing DMPs and new DMPs for complex medical patients.

In this study, the most common combination among patients with 2 + PDs was the type 2 diabetes with the cardiovascular PD, with a prevalence of 22.5%. The association of type 2 diabetes and cardiovascular disease is well known (Marks and Raskin 2000); thus, it would be reasonable to suggest a DMP for patients who have both conditions. Although we found that 40.4% of these patients had at least one additional PD, this result indicates that multimorbidity may consist of many disease combinations in different patterns, supporting other international research (Prados-Torres et al. 2014). To develop DMPs only for the most prevalent pairs of PDs is therefore not a total solution.

Patients with 2 + PDs had a high mortality risk and a high risk of being acutely hospitalized 6 months prior to the index admission and of being readmitted within 90 days from discharge. Several studies have found that healthcare costs rise as patients develop more than one chronic disease (Nagl et al. 2012; Foguet-Boreu et al. 2014; Moffat and Mercer 2015; Palladino et al. 2016). Fragmented care based on treating single diseases in isolation can lead to duplication of treatment (Barnett et al. 2012; Salisbury 2012) and hence higher costs. Patients with multimorbidity do differ; however, a study from The Netherlands showed that the majority of patients with multimorbidity did not have a higher level of healthcare costs than patients with only on chronic disease, but that a small group of patients with multimorbidity had a very high level of costs (Hopman et al. 2015). These patients were older, female, had low income and suffered from more chronic diseases (Hopman et al. 2015).

Patients with the cancer PD had the highest median LOS, high mortality and readmission rates within 7 and 90 days, and the lowest proportion of patients with 2 + PDs compared to patients belonging to the other PDs, resulting in a low degree of complexity. This result indicates an advantage in keeping a single disease perspective for older patients with cancer.

We found that 41.9% of the patients did not have a PD and were therefore not eligible to enter a DMP. In this group, 88.1% had been hospitalized or examined in outpatient clinics with diseases in two or more ICD-10 disease categories, possibly reflecting the burden and complexity of the patient’ conditions and the degree of multimorbidity. Furthermore, we found that patients without a PD had a cumulative probability of 28.8% of being readmitted within 90 days, which was significantly lower than for patients with a PD, though still relatively high. Despite this complexity, patients without a PD had a LOS of only 1 day, risking fragmented care. Hence, focusing on a few chronical diseases does not solve the challenges for the older medical patients. A solution could be to use measures of frailty, which is found to be associated with an increased risk of disability, hospitalization and long-term care (Clegg et al. 2013), in combination with disease patterns as an indicator of the need for care management. Frailty in combination with disease patterns could help hospitals communicate to the primary care sector about which patients are in need of structured care to prevent hospitalizations and readmissions.

A strength of this study is its population-based nature, made possible by the Danish CPR number system, allowing linkage at the individual level across nationwide and local registries. We used a 10-year prevalence to define whether a patient belonged to a PD, as recommended by Schram et al. (2008). Furthermore, this study covered patients both living at home and in care institutions.

Our study also has limitations. First, it is likely that the registration of secondary diagnoses is focused on those relevant for the specific hospital admission and thus does not necessarily reflect all secondary diagnoses for the patient. Furthermore, the diagnoses are based on routine discharge registration and it cannot be excluded that physicians differ with regard to coding quality possibly introducing a risk of miscoding and undercoding. However, a recent study by Thygesen et al. (2011) found that the positive predictive value of ICD-10 codes used to assess the Charlson comorbidity index score was 98% in the Danish National Patient Register. By including ICD-10 codes from both hospitalizations and outpatient visits from 2002 to 2012, we have tried to increase the validity of the diagnosis codes. Second, the method used for data collection in this study was register based. Schram et al. (2008) found that the setting characteristics have an important influence on the outcome of multimorbidity, with multimorbidity being more prevalent in a general practice setting than in a hospital setting. Hence, the prevalence of multimorbidity may be underestimated in this study. Third, the inclusion of only one hospital may have reduced the generalizability of the results, though Amager and Hvidovre Hospital covers 10 different municipalities with different socioeconomic levels, and we therefore believe that the results are reasonably representative.

In conclusion, we found that overlaps of PDs defined by the DMPs appeared in many combinations with mostly low prevalence among acutely hospitalized older medical patients. Patients with 2 + PD had the highest risk of readmission; however, patients without a PD still had a cumulative risk of 28.8% for readmission within 90 days. Hence, patients without a PD as well as patients with 2 + PD are complex groups that could stand to benefit from a more holistic approach in designing DMPs. However, for patients with cancer, keeping a single disease perspective may be advantageous.
